# Botulinum Toxin Injections for Drooling Improve Dysphagia in Patients with Parkinson’s Disease

**DOI:** 10.3390/toxins18020073

**Published:** 2026-02-02

**Authors:** Domenico Antonio Restivo, Mario Stampanoni Bassi, Angelo Alito, Simona Portaro, Adriana Tisano, Salvatore Greco, Rosario Marchese-Ragona, Angelo Quartarone

**Affiliations:** 1 Department of Clinical and Experimental Medicine, University of Messina, 98125 Messina, Italy; atisano@unime.it; 2 Brain Mapping Lab, Department of Biomedical, Dental Sciences and Morphological and Functional Imaging, University of Messina, 98125 Messina, Italy; 3 Unit of Neurology, IRCCS Neuromed, 86077 Pozzilli, Italy; 4 Faculty of Psychology, Uninettuno Telematic international University, 00186 Rome, Italy; 5 Department of Biomedical, Dental Sciences and Morphological and Functional Images, University of Messina, 98125 Messina, Italy; alitoa@unime.it; 6 Physical Rehabilitation Medicine Department, University Hospital “G. Martino”, 98125 Messina, Italy; simonaportaro@hotmail.it; 7 Neurological Unit, ARNAS “Garibaldi”, 95123 Catania, Italy; salvogreco73@gmail.com; 8 Department of Neurosciences, Otolaryngology Section, University of Padova, 35122 Padova, Italy; rosario.marcheseragona@unipd.it; 9 IRCCS Centro Neurolesi Bonino Pulejo, 98124 Messina, Italy; angelo.quartarone@irccsme.it

**Keywords:** botulinum toxin, dysphagia, drooling, Parkinson’s disease, swallowing

## Abstract

Drooling and dysphagia are frequent and disabling complications in Parkinson’s disease (PD) and often coexist, with drooling mainly resulting from impaired saliva clearance due to reduced oral motor control and potentially worsening swallowing function. This study aimed to evaluate whether botulinum toxin type A (BoNT/A) injections into the major salivary glands, beyond controlling drooling, could also improve swallowing performance using clinical and neurophysiological measures. Twenty PD patients with severe drooling and dysphagia underwent bilateral ultrasound-guided BoNT/A injections into the parotid and submandibular glands. Assessments were performed at baseline and at 1, 8, and 12 weeks post-injection. Dysphagia severity was evaluated using the Penetration–Aspiration Scale and the Dysphagia Severity Rating Scale. Neurophysiological assessment included electromyographic recordings from suprahyoid/submental and cricopharyngeal muscles, together with mechanomyography analysis of laryngeal movement during swallowing. Following BoNT/A treatment, a consistent reduction in drooling was observed, accompanied by significant improvements in clinical dysphagia scores and neurophysiological swallowing parameters across all follow-up time points. These findings suggest that incobotulinumtoxinA injections into salivary glands not only reduce drooling but also enhance swallowing function in PD patients, possibly by facilitating oral floor and oropharyngeal motor coordination secondary to improved saliva management.

## 1. Introduction

Among the non-motor manifestations of Parkinson’s Disease (PD), sialorrhea (drooling) and dysphagia (difficulty swallowing) are particularly disabling complications that often occur together in severe stages of the disease [1,2,3]. These conditions have a significant impact on patients’ quality of life and increase the risk of aspiration pneumonia, a leading cause of death in patients with advanced PD [1,3]. The prevalence of dysphagia in PD is estimated to be between 40% and 80% [4]. Furthermore, dysphagia has been shown to have a negative impact on the quality of life of affected patients due to progressive impairment of oral intake, resulting in dehydration, weight loss, malnutrition, and limitations in social activities [5].

In PD, all three phases of swallowing may be affected and can occur at any stage of the disease. While swallowing difficulties are more evident in advanced stages, they may be present in earlier stages in a few patients [6]. Furthermore, dysphagia often worsens concurrently with disease progression [3,7]. Although both classical dopaminergic drugs and advanced treatments, including infusions and deep brain stimulation, have been shown to be effective in treating motor symptoms, the effectiveness of these therapies in addressing swallowing disturbances is controversial [8,9,10].

The most practiced therapies for dysphagia in PD aim to improve swallowing safety and efficiency symptomatically by means of compensatory and rehabilitative strategies [11]. These treatments aim to reduce morbidity and mortality associated with malnutrition and aspiration pneumonia. Neurostimulation techniques have been proposed as an adjunct to conventional swallowing therapy [12]. Recently, a consensus panel statement has been developed to provide guidance on multidisciplinary treatment of dysphagia in PD patients [13].

Injecting botulinum toxin (BoNT) into the cricopharyngeal (CP) muscle of the upper esophageal sphincter (UES) has been shown to improve dysphagia in some PD patients with pharyngeal-phase involvement associated with CP muscle hyperactivity in advanced disease stages [14].

On the other hand, approximately 70–80% of patients with PD are affected by sialorrhea. It primarily results from slowed saliva clearance due to reduced oral movement, rather than excessive saliva production. The accumulation of saliva in the oral cavity leads to drooling. Other than causing social embarrassment, this may also worsen dysphagia, consequently increasing the risk of aspiration in PD patients [2,13,15]. However, the relationship between drooling and dysphagia in PD remains incompletely understood.

While both conditions share common pathophysiological mechanisms related to bradykinesia and rigidity of the oropharyngeal muscles, the specific impact of excessive saliva accumulation on swallowing function has not been thoroughly investigated. The constant presence of pooled saliva in the oral cavity and pharynx may overwhelm compromised swallowing mechanisms, leading to increased penetration and aspiration events [16]. Furthermore, the volume and consistency of saliva may affect the sensory feedback necessary for triggering appropriate swallowing responses, potentially worsening dysphagia severity. It has been demonstrated that drooling increases the risk of aspiration and aspiration pneumonia in PD patients [13,15]. Nevertheless, it is still unclear whether severe drooling directly exacerbates dysphagia.

However, it is likely that reduced orofacial praxis is involved. Additionally, there is no definitive answer as to whether reducing sialorrhea and/or drooling through specific therapeutic interventions could improve swallowing function. This knowledge gap represents a significant limitation in our understanding of the relationship between these two conditions and may have important implications for treatment strategies.

BoNT into the major salivary glands (the parotid and submandibular glands) have emerged as an effective treatment for sialorrhea and drooling in patients with PD. BoNT works by blocking the release of acetylcholine at the glandular level, thereby reducing saliva production. Several studies have confirmed the efficacy and safety of this treatment in reducing drooling associated with neurological disorders [14,17,18,19,20].

Given the proven effectiveness of BoNT in treating drooling, and the close relationship between saliva management and swallowing function, we hypothesized that reducing drooling with BoNT injections could improve dysphagia.

This study aimed to evaluate the effects of BoNT injections into the major salivary glands on swallowing performance in patients with Parkinson’s disease who presented with severe drooling and dysphagia. To this end, we conducted a longitudinal study, assessing swallowing function both before and after treatment, using validated clinical scales and objective neurophysiological measures.

## 2. Results

The study included twenty patients (12 males and 8 females with a mean age of 68.1 ± 3.8 years). The mean disease duration was 12 ± 2.2 years, and the mean Hoehn and Yahr stage was 3.4 ± 0.6 [21]. The demographic and clinical characteristics of the study population are summarized in Table 1.

All patients received stable dopaminergic therapy throughout the study. Prior to treatment, videofluoroscopy revealed reduced pharyngeal clearance and incomplete opening of the CP muscle in all patients. At baseline, the mean scores on the Penetration-Aspiration Scale (PAS) [22], the Dysphagia Severity Rating Scale (DSRS) [23] and the Drooling Frequency and Severity Scale (DFSS) [24] were 5.65 ± 0.81, 4.65 ± 0.87 and 7.3 ± 0.8, respectively. Laryngeal transit time duration (LTE-D) was abnormal in 19 out of 20 patients (95%). Abnormal results were seen in the submental hyoid EMG leading edge (SHEMG-LE) interval in 18 patients (90%), the submental hyoid EMG duration (SHEMG-D) interval in 14 patients (70%), and the cricopharyngeal EMG duration (CPEMG-D) interval in 12 patients (60%) [25,26].

### 2.1. Outcome Measures

Significant improvements were observed in both the primary and secondary outcome measures following botulinum toxin treatment, across all three evaluation sessions. This demonstrates a clinically meaningful improvement in overall dysphagia severity. Videofluoroscopy (VFS) also revealed notable enhancements in pharyngeal clearance and CP muscle opening after BoNT/A treatment.

#### 2.1.1. Primary Outcome

At baseline, the mean PAS score was 5.65 ± 0.81. A PAS score of less than 6 was observed in 11 patients (55%), indicating moderate dysphagia. Conversely, a score greater than 6 was observed in the remaining nine patients (45%), indicating more severe dysphagia. Applying the Wilcoxon signed-rank test to the mean PAS values revealed a substantial reduction in penetration and aspiration events, with highly significant differences observed between baseline and each post-treatment period (*p* < 0.001). A post hoc analysis confirmed significant differences between baseline and each post-treatment session (*p* < 0.001). All numerical results are summarized in Table 2.

#### 2.1.2. Secondary Outcomes

The mean DSRS score improved significantly from baseline throughout the evaluation sessions (*p* < 0.001). The pre- and post-treatment DSRS scores are reported in Table 2. Statistically significant improvements in all measured neurophysiological parameters following botulinum toxin treatment indicate enhanced coordination and efficiency of the swallowing mechanism. This effect was most notable at weeks 1 and 8 but remained significant at week 12. The differences between the baseline and the post-treatment periods were statistically significant for all parameters. ANOVA revealed significant changes in the secondary outcome measures (*p* < 0.001). A post hoc analysis confirmed that the differences between baseline and each post-treatment session were also highly significant for the secondary outcome measures (*p* < 0.001). Pre- and post-treatment LT-D, SHEMG-LE, SHEMG-D and CPEMG-D values are reported in Table 2.

## 3. Discussion

Dysphagia and drooling can very often complicate PD [1,2,3]. These two pathological conditions often coexist, particularly in advanced stages of the disease. However, beyond their occurrence in PD, it is unclear how these conditions interact and whether sialorrhea can induce or exacerbate dysphagia in these patients, and if so, what the underlying mechanism is. BoNT injections have been shown to be safe and effective in treating sialorrhea and drooling associated with PD [14,18,19,20,27]. There is no data on whether the efficacy of this treatment in reducing drooling may also improve swallowing performance.

In this study, we evaluated the effectiveness of BoNT/A injections into the major salivary glands in controlling drooling and improving dysphagia in PD, as measured by clinical and neurophysiological methods.

We demonstrated that, in addition to improving salivary control, BoNT/A injection into the salivary gland resulted in a significant improvement in primary and secondary swallowing outcomes in patients with PD.

The improvement induced by BoNT/A was evident in all the clinical, instrumental, and electrophysiological measures evaluated. The substantial reduction in mean PAS and DSRS indicates that reducing drooling reduces the risk of aspiration pneumonia. This effect was evident at week 1 and persisted at weeks 8 and 12 after treatment. Maximal efficacy occurred at week 1 and persisted throughout the subsequent evaluation sessions. The effect of BoNT/A treatment on dysphagia following sialorrhea can be explained by several interacting mechanisms. Firstly, reducing saliva volume decreases the baseline liquid load in the oral cavity and pharynx, thereby reducing the risk of overflow aspiration and enabling the compromised swallowing mechanism to function more effectively [28,29].

In patients with PD who have bradykinetic and poorly coordinated swallowing, even normal saliva production may exceed the capacity for safe clearance [30]. Reducing this volume brings the salivary load within the patient’s swallowing capacity. Secondly, excessive saliva pooling may interfere with the sensory feedback mechanisms that are crucial for triggering and coordinating the swallowing reflex [31]. The constant presence of saliva in the pharynx may lead to sensory adaptation, reducing the sensitivity of the mechanoreceptors and chemoreceptors that normally initiate protective swallowing responses [32,33,34]. By reducing saliva volume, BoNT/A treatment can restore more physiological sensory conditions and allow for more appropriate triggering and coordination of swallowing. Improvement in the efficacy of sensory inputs is documented by improvements in neurophysiological measures, particularly the SHEMG-LE and the LT-D. Improvement in SHEMG-LE indicates that BoNT/A reduces saliva and enhances the swallowing reaction time, improving the velocity with which the oral phase of swallowing is initiated.

Improvement in LT-D parameters indicates an improvement in the velocity of the overall swallowing act. The reduction in SHEMG-D observed after BoNT/A treatment suggests that it significantly improves the endurance of the oral floor muscles involved in the oral phase of swallowing. These changes inevitably lead to an increase in the duration of the CP muscle pause during the pharyngeal phase of swallowing.

Although CP muscle activity is involved in the pharyngeal phase of swallowing, CP muscle relaxation is also closely linked to the activity of the submental muscle, which elevates the hyoid bone during swallowing [25,35,36].

Furthermore, the overall neurophysiological improvements observed in this study suggest enhanced efficiency and coordination of the swallowing motor program. These improvements may reflect the mechanical benefit of reduced saliva volume, as well as potential neuroplastic changes in swallowing control. Reduced sensory overload from excessive saliva may enable more effective cortical and subcortical processing of swallowing-related information, resulting in improved motor output [37].

The present findings suggest that BoNT for sialorrhea may have a beneficial effect on swallowing function via multiple interacting mechanisms. By reducing saliva volume, BoNT/A decreases the baseline liquid load in the oral cavity and pharynx. This lowers the risk of penetration or aspiration related to overflow and facilitates more efficient oral and pharyngeal motor coordination. These physiological changes are supported by improvements observed in both clinical dysphagia scales and objective neurophysiological parameters, including enhanced timing and coordination of the swallowing musculature.

However, several issues remain to be clarified. Firstly, the optimal dosage and injection strategy required to maximize the benefits for both drooling and dysphagia have yet to be defined. Although a standardized dosing protocol for sialorrhea was adopted in this study, alternative doses or glandular targeting may further optimize swallowing outcomes. Secondly, the potential synergistic effects of combining BoNT treatment with other dysphagia-directed interventions, such as behavioral swallowing therapy or neuromodulatory approaches, warrant further investigation. Therefore, larger, controlled studies are needed to confirm these findings and establish integrated treatment strategies for patients with PD.

## 4. Conclusions and Future Perspectives

This preliminary study provides evidence for the first time that BoNT treatment for severe drooling in PD is associated with significant improvements in dysphagia, as demonstrated by clinical scales and objective neurophysiological measures. As well as reducing excessive salivation, BoNT/A injections into the major salivary glands appear to improve the coordination of the swallowing musculature, enhancing swallowing safety and efficiency and reducing penetration and aspiration events. Probably as a consequence of the improved oral phase coordination and speed, this improvement involves also the performance of the pharyngeal muscle leading to a more effective UES relaxation. Our findings suggest that sialorrhea management should be considered not only as a treatment for drooling, but also as a relevant therapeutic strategy in the comprehensive care of dysphagia in PD.

### Study Limitations and Future Perspectives

The major limitation of this study is the small number of involved patients for this reason it should be considered as a pilot study. Further studies on lager PD patient population eventually also evaluating the effect of association of salivary gland injections with swallowing rehabilitation maneuvers are needed to confirm the results of this preliminary study.

## 5. Materials and Methods

### 5.1. Study Design and Participants

This longitudinal observational pilot study was retrospectively conducted on twenty consecutive patients with PD-associated dysphagia and sialorrhea, or drooling, who were referred to the Department of Neurology at the ARNAS “Garibaldi” in Catania, Italy, between May 2022 and December 2024, by using our patient database.

The study was approved by the Ethics Committee Catania 2 (CECT2) (minutes No. 87/2022/CECT2, 18 January 2022) and was conducted in accordance with the Declaration of Helsinki. All patients provided written informed consent prior to participation.

The inclusion criteria were: (1) a clinically established diagnosis of idiopathic PD according to the Movement Disorder Society (MDS) diagnostic criteria [38], confirmed by a movement disorders specialist; (2) the presence of sialorrhea or drooling requiring treatment within the previous three months; (3) documented dysphagia, confirmed by clinical assessment and instrumental evaluation; (4) a modified Hoehn and Yahr stage of ≤4 and the ability to stand without assistance; (5) antiparkinsonian medication (levodopa or dopamine agonists) for at least four weeks prior to enrolment; and (6) the ability to provide informed consent. Exclusion criteria included: (1) previous treatment with botulinum toxin for sialorrhea within the previous six months; (2) contraindications to BoNT therapy; (3) the presence of structural abnormalities of the oropharynx or esophagus; (4) a history of head and neck surgery or radiotherapy; (5) other neurological conditions affecting swallowing; and (6) severe cognitive impairment precluding cooperation with assessments.

### 5.2. Outcome Measures

Patients were prevented from eating or drinking for one hour prior to each assessment. Drooling and swallowing were evaluated at baseline (week 0) and at 1, 4 and 8 weeks following the BoNT injection into the major salivary glands. This was performed using multiple outcome measures, including clinical scales and neurophysiological parameters.

#### 5.2.1. Drooling Frequency and Severity Scale

Drooling was evaluated using the DFSS. This 9-point scale evaluates the frequency (5 points) and severity (4 points) of drooling. Frequency score: 1 = Dry: never drools; 2 = Mild: lips only are wet; 3 = Moderate: lips and chin are wet; 4 = Severe: drools to the extent that clothing becomes damp; 5 = Profuse: clothing, hands, tray, and objects become wet. The severity score ranges from 1 (never drools) to 5 (constantly drools) [24].

#### 5.2.2. Swallowing Evaluation

Swallowing function was evaluated using instrumental and clinical tools. The instrumental measures included radiological evaluations (VFL) and electromyographic recordings of the muscles involved in oropharyngeal swallowing. The clinical measures included the PAS which is a scale for the semi-quantitative assessment of penetration and/or aspiration phenomena) and the DSRS, both of which were evaluated based on VFS results [22,23].

#### 5.2.3. Videofluoroscopy

VFS with modified barium swallowing was performed using a standard radiographic fluoroscopic system with a remote monitor and videofluoroscopic capabilities. Images were acquired in real time at 30 frames per second (fps) and recorded by a VHS digital video recorder at 25 fps. The data were stored on a mini digital video tape and reviewed frame by frame. All subjects were instructed to retain the full volume in their mouth until asked to swallow. Images were taken in the lateral view; the anatomical markers for imaging were the lips anteriorly, the cervical spine posteriorly, the nasopharynx superiorly and the upper margin of the thoracic esophagus inferiorly. Three different food consistencies of standardized bolus size were used: thin liquid (equivalent to milk), semi-solid (equivalent to jelly) and solid (dry toast coated in barium).

#### 5.2.4. Penetration-Aspiration Scale

The results of all videofluoroscopic swallowing examinations were evaluated by a speech and language pathologist using the PAS, an 8-point scale for the semi-quantitative assessment of airway penetration and aspiration based on endoscopic and/or radiological findings. PAS scores are defined as follows: 1, material does not enter the airway; 2, material enters the airway, remains above the vocal folds, and is ejected; 3, material enters the airway, remains above the vocal folds, and is not ejected; 4, material enters the airway, contacts the vocal folds, and is ejected; 5, material enters the airway, contacts the vocal folds, and is not ejected; 6, material enters the airway, passes below the vocal folds, and is ejected into the larynx or out of the airway; 7, material enters the airway, passes below the vocal folds, and is not ejected from the trachea despite effort; and 8, material enters the airway, passes below the vocal folds, and no effort is made to eject the material [22].

#### 5.2.5. Dysphagia Severity Rating Scale

Dysphagia severity was clinically evaluated using the DSRS, a 7-point scale that grades swallowing impairment from 0 to 6. The scale is defined as follows: 0, normal swallowing; 1, minimal dysphagia, with instrumental examination showing slight deviation from normal swallowing and possible patient-reported changes in swallowing sensation, without the need for dietary modification; 2, mild dysphagia, characterized by oropharyngeal dysphagia manageable with specific swallowing strategies and possible slight modification of diet consistency; 3, mild-to-moderate dysphagia, with a potential risk of aspiration that can be reduced through specific swallowing techniques and a modified diet, often associated with significantly prolonged mealtimes and possible need for supplemental nutrition; 4, moderate dysphagia, with a significant risk of aspiration and minimal signs of aspiration of one or more consistencies on instrumental examination, requiring specific techniques, supervision during meals, and possible oral or enteral nutritional supplementation; 5, moderately severe dysphagia, in which the patient aspirates 5–10% of one or more consistencies with potential aspiration across all consistencies, often associated with an absent or non-protective cough reflex and requiring alternative feeding methods to maintain adequate nutrition, with “nothing by mouth” indicated if pulmonary status is compromised; and 6, severe dysphagia, defined by aspiration greater than 10% for all consistencies, for which “nothing by mouth” is recommended [39].

#### 5.2.6. Neurophysiological Parameters

Simultaneous EMG recordings were performed from three channels while patients swallowed a 5 mL water bolus from a syringe. The first channel used a piezoelectric transducer (mechanomyogram) positioned over the thyroid cartilage to monitor the whole laryngeal excursion movements (elevation and relocation) during voluntary swallowing. The second channel involved surface electrode recordings of the EMG activity of the suprahyoid/submental muscle complex (consisting of the mylohyoid, genioglossus, and ventral belly of the digastric muscle), which were applied to the skin of the suprahyoid region (with an interelectrode distance of 30 mm). This activity marks the beginning of the propulsive action of the tongue during the oral phase of swallowing, continuing throughout the pharyngeal phase. The third channel was used to record the EMG activity of the CP muscle and of the UES using a concentric needle electrode inserted through the skin at the level of the cricoid cartilage, 1.5 cm posterior to its palpable lateral border in a posteromedial direction [23,25]. At rest, the muscle exhibits tonic involuntary activity related to its function as a muscular sphincter. This activity completely disappears for a brief period (approximately 0.6–1 s) during the pharyngeal (hypopharyngeal) phase of swallowing. CP muscle inhibition (relaxation) allows the bolus to transit into the upper esophageal tract. For CP recordings, the EMG needle was inserted 1.5 cm lateral to the palpable lateral border of the cricoid cartilage with a posteromedial direction. In physiological conditions, a high-frequency tonic EMG activity can be observed at rest as the needle penetrates the muscle. Conversely, an electromyographic of approximately 500–1000 ms occurs on voluntary swallowing [35,36,40,41]. The absence or reduction (duration < 500 ms) of the CP inhibition on voluntary swallowing were considered indicative of UES hyperactivity/dysfunction.

EMG signals were band-pass filtered between 100 Hz and 2 kHz, while the piezoelectric transducer signal was band-pass filtered between 0.01 and 30 Hz. The signal sampling rate was 20 Hz for each channel. Myoelectric activity signals were passed through a preamplifier (CED 1902, Cambridge Electronic Design, Cambridge, UK) and then collected via a laboratory interface (CED 1401). Each patient was examined while seated with their head in a natural position. They were then asked to swallow a small volume of water (5 mL), which was introduced into their mouth with a disposable syringe without a needle. The following neurophysiological measures were analyzed: (1) Duration of laryngeal transducer excursion (LTE-D); (2) Duration of EMG activity of suprahyoid/submental muscles (SHEMG-D); (3) Duration of CP muscle inhibition (CPEMG-D); (4) Interval between onset of EMG activity of suprahyoid/submental muscles and onset of laryngeal elevation (SHEMG-LE). The signals were triggered when the suprahyoid/submental muscle EMG activity signal amplitude was equal to or greater than 50 mV. Recordings obtained from ten successive swallows were stored and the average value of each electrophysiological measure was calculated [23,25].

The primary outcome of the study was the change in the mean PAS score from baseline to each post-treatment assessment, reflecting modifications in swallowing safety. Secondary outcomes included changes in the mean DFSS and DSRS scores, as well as in neurophysiological swallowing parameters, namely LTE-D, SHEMG-D, SHEMG-LE, and CPEMG-D.

### 5.3. Intervention

At baseline, each patient received ultrasound-guided injections of botulinum toxin type A into the major salivary glands, using incobotulinumtoxinA (Xeomin^®^, Merz Pharmaceuticals, Frankfurt, Germany), diluted in 2 mL of 0.9% saline solution. For each patient, two parotid and two submandibular glands were injected. For gland localization, the probe was positioned over the anatomical landmarks. For parotid gland it corresponds to the approximately midline point between the tragus and the angle mandible one finger’s breadth inside. The anatomical landmark for submandibular gland corresponds approximately to the midline point between the angle of mandibula and the chin extremity one finger’s breadth. Two injections for each gland were at two different points about 3 cm apart. A dose of 30 units was injected into each parotid gland and 15 units into each submandibular gland, resulting in a total dose of 90 units per patient per treatment session. All injections were performed with a 27-gauge needle under real-time ultrasound guidance to ensure accurate intraglandular placement and were administered by the same experienced clinician.

### 5.4. Statistical Analysis

Pre- and post-treatment data were compared using parametric or non-parametric statistical analyses according to data distribution, as assessed by the Shapiro–Wilk test. Normality testing was performed with a significance threshold of *p* < 0.001. To evaluate changes in swallowing-related outcome measures across the post-treatment follow-up periods, repeated-measures analysis of variance (ANOVA) was applied for normally distributed variables, whereas the Wilcoxon signed-rank test was used for non-normally distributed data. When significant main effects were detected, post hoc pairwise comparisons between baseline and each post-treatment time point were conducted using *t*-tests with Bonferroni correction. For all statistical comparisons, the level of significance was set at *p* < 0.05. Statistical analyses were performed using SYSTAT software, version 11 (Systat Inc., Evanston, IL, USA).

## Figures and Tables

**Table 1:** Demographic and clinical characteristics of patients with Parkinson’s disease included in the study. Data are presented as mean ± standard deviation.

Age	H/Y	Disease Duration	PAS (W0)	DFSS (W0)	DSRS (W0)	LTE-D (W0)
68	3	8	5	7	4	778
67	3	13	5	7	4	774
69	3	14	5	7	5	678
70	3	13	6	7	5	543
71	3	14	5	7	4	266
65	4	12	5	8	4	786
77	3	15	5	6	4	460
70	3	15	6	7	4	665
64	3	13	5	6	3	720
66	3	14	6	7	5	703
65	3	12	5	8	4	256
68	5	10	5	7	5	678
69	3	10	6	7	5	543
71	3	12	7	9	6	501
73	3	9	7	8	6	786
63	5	12	7	9	6	604
73	3	11	5	7	4	706
61	3	9	7	8	6	810
65	3	13	6	7	5	665
69	3	15	5	7	4	720
68.2 ± 3.84	3.25 ± 0.63	12.2 ± 2.11	5.65 ± 0.81	7.3 ± 0.80	4.65 ± 0.87	636.25 ± 157.58

PAS = Penetration–Aspiration Scale; DSRS = Dysphagia Severity Rating Scale; DFSS = Drooling Frequency and Severity Scale; LTE-D = Laryngeal Transit Time Duration; H/Y = Hoehn and Yahr.

**Table 2:** Clinical and neurophysiological swallowing outcomes at baseline and after botulinum toxin treatment in patients with Parkinson’s disease. Data are reported as mean ± standard deviation.

	Week 0	Week 1	Week 4	Week 8
PAS	5.65 ± 0.81	3.2 ± 1.82	3 ± 1.97	3.45 ± 1.95
DSRS	4.65 ± 0.87	2.35 ± 1.63	2.3 ± 1.67	2.4 ± 1.72
DFSS	7.3 ± 0.8	2.75 ± 1.74	2.6 ± 1.75	2.7 ± 1.62
LTE-D	635.25 ± 157.58	558.71 ± 136.71	562.33 ± 137.95	585.38 ± 145.6
SHEMG-LE	2141 ± 343.11	1928.9 ± 369.21	1999.61 ± 347.61	2023.47 ± 347.94
SHEMG-D	1774.04 ± 614.41	1612 ± 623.79	1650.47 ± 635.28	1683.28 ± 646.48
CPEMG-D	431.14 ± 140,06	493.9 ± 54.97	495.52 ± 53.94	496.42 ± 54.38

PAS = Penetration–Aspiration Scale; DSRS = Dysphagia Severity Rating Scale; DFSS = Drooling Frequency and Severity Scale; LTE-D = Laryngeal Transit Time Duration; SHEMG-LE = Submental–Hyoid Electromyographic Leading Edge; SHEMG-D = Submental–Hyoid Electromyographic Duration; CPEMG-D = Cricopharyngeal Electromyographic Duration.

## Data Availability

The original contributions presented in this study are included in the article. Further inquiries can be directed to the corresponding author.

## Abbreviations

The following abbreviations are used in this manuscript:

BoNT	Botulinum toxin
CP	Cricopharyngeal
CPEMG-D	Cricopharyngeal EMG duration
DFSS	Drooling Frequency and Severity Scale
DSRS	Dysphagia Severity Rating Scale
EMG	Electromyography
LTE-D	Laryngeal transit time duration
MDS	Movement Disorder Society
PAS	Penetration-Aspiration Scale
PD	Parkinson’s disease
SHEMG-LE	Submental hyoid EMG leading edge
UES	Upper esophageal sphincter
VFS	Videofluoroscopy
